# Identification and characterization of two types of amino acid-regulated acetyltransferases in actinobacteria

**DOI:** 10.1042/BSR20170157

**Published:** 2017-07-04

**Authors:** Yu-Xing Lu, Xin-Xin Liu, Wei-Bing Liu, Bang-Ce Ye

**Affiliations:** 1 Lab of Biosystems and Microanalysis, Biomedical Nanotechnology Center, State Key Laboratory of Bioreactor Engineering, East China University of Science and Technology, Shanghai 200237, China; 2 School of Chemistry and Chemical Engineering, Shihezi University, Xinjiang 832000, China

**Keywords:** allosteric regulation, ACT domain, protein acetyltransferase, protein acetylation

## Abstract

One hundred and fifty GCN5-like acetyltransferases with amino acid-binding (ACT)-GCN5-related *N*-acetyltransferase (GNAT) domain organization have been identified in actinobacteria. The ACT domain is fused to the GNAT domain, conferring amino acid-induced allosteric regulation to these protein acetyltransferases (Pat) (amino acid sensing acetyltransferase, (AAPatA)). Members of the AAPatA family share similar secondary structure and are divided into two groups based on the allosteric ligands of the ACT domain: the asparagine (Asn)-activated PatA and the cysteine (Cys)-activated PatA. The former are mainly found in *Streptomyces*; the latter are distributed in other actinobacteria. We investigated the effect of Asn and Cys on the acetylation activity of Sven_0867 (*Sve*PatA, from *Streptomyces venezuelae* DSM 40230) and Amir_5672 (*Ami*PatA, from *Actinosynnema mirum* strain DSM 43827), respectively, as well as the relationship between the structure and function of these enzymes. These findings indicate that the activity of PatA and acetylation level of proteins may be closely correlated with intracellular concentrations of Asn and Cys in actinobacteria. Amino acid-sensing signal transduction in acetyltransferases may be a mechanism that regulates protein acetylation in response to nutrient availability. Future work examining the relationship between protein acetylation and amino acid metabolism will broaden our understanding of post-translational modifications (PTMs) in feedback regulation.

## Introduction

Protein lysine acetylation has emerged as an important metabolic regulatory mechanism in bacteria, since the discovery of reversible acetylation of acetyl–CoA (Ac–CoA) synthetase via enzymatic acetylation, such as acetyltransferase [1] and deacetylase [2]. Protein acetylation also can occur via nonenzymatic reaction, such as chemical acetylation, wherein the intracellular acetyl-phosphate (AcP) plays a critical role [3,4]. The intracellular level of protein acetylation is ultimately defined by the concentration of AcP (or Ac–CoA) and relative activities of acetyltransferases and deacetylases. These two classes of enzymes respond to intracellular nutritional status to control the acetylation of specific proteins that mold the metabolic network [5,6]. Multiple nutrient-mediated signaling pathways regulate the transcription of genes encoding acetyltransferases and deacetylases. In *Bacillus subtili*s, regulating the expression of the *acuA* gene, which encodes an acetyltransferase, is under the control of catabolite control protein A (CcpA), a global regulatory protein affected by the quality of the carbon source available to the cell [5]. In *Escherichia coli*, two acetylation-signaling pathways mediated by cAMP have been identified. The carbon regulating CRP–cAMP complex induces the expression of *yfiQ*, which encodes the Gcn5-like acetyltransferase YfiQ (also known as PatZ and Pka) [7,8], thus increasing the acetylation level of proteins in response to the intracellular cAMP signal [6]. Furthermore, cAMP–CRP regulates the glucose-induced, AcP-dependent protein acetylation in response to carbon overflow, with specificity of the acetylation determined by the accessibility, reactivity, and 3D microenvironment of the target lysine [9]. In *Salmonella enterica*, the expression of the genes encoding the acetyltransferase Pat and deacetylase CobB is activated by IolR, a repressor of *myo*-inositol catabolism involved in the utilization of cyclohexane-1,2,3,4,5,6-hexol, a cyclic polyol abundant in soil, as a carbon source [10].

Allosteric effectors also control the activity of protein acetyltransferases (Pat). In *Mycobacterium tuberculosis* and *Mycobacterium smegmatis*, cAMP directly activates the Pat, *Mt*Pat (Rv0998) and *Ms*Pat (MSMEG_5458) by binding to a cyclic nt-binding domain that is fused to the N-terminus of the catalytic GCN5-related *N*-acetyltransferase (GNAT) domain [11]. Recently, we found that an ACT domain, fused to the GNAT acetyltransferase of *Micromonospora aurantiaca* (*Ma*Kat), is used for amino acid-induced allosteric regulation of the enzyme [12]. In the present study, we identified a total of 150 putative amino acid sensing acetyltransferases (AAPatA) that have an ACT-GNAT domain organization specific to actinobacteria through bioinformatics analysis. The ACT domain is fused to the GNAT domain, conferring an amino acid-induced allosteric effect to the regulation of protein acetylation. We found that members of the AAPatA family are divided into two groups: the asparagine (Asn)-regulated PatA and cysteine (Cys)-regulated PatA. The former exists mainly in *Streptomyces*; the latter are distributed in other actinobacteria. Our results present an example of a novel signal transduction mechanism for regulating protein acetylation in actinobacteria via amino acid-sensing acetyltransferases in response to intracellular concentrations of Asn and Cys.

## Materials and methods

### Strains and reagents

All strains were purchased from the China General Microbiological Culture Collection Center (CGMCC). All the reagents, purchased from Sigma, were of the highest grade available commercially.

### Construction of plasmids and overexpression and purification of proteins

All *patA* and homologous *acs* genes coding for Ac–CoA synthetase were amplified by PCR using genomic DNA from bacterial strains. After digestion with restriction enzymes, genes coding for PatA and Ac–CoA synthetase (Acs) proteins were ligated into pET-28a, which was predigested with EcoRI and HindIII, generating pET28a-PatA and pET28a-Acs. The overexpression and purification of proteins in the *E. coli* BL21-DE3 was performed as described previously [12]. Protein concentration was determined using the BCA method.

### Acetylation activity and steady-state kinetic assay of PatA acetyltransferases

Acetylation activity of acetyltransferases was measured continuously by a coupled enzymatic assay using a fluorescence plate reader (BioTek Instruments, Winooski, U.S.A.). The production of CoA is measured using pyruvate dehydrogenase mediated reduction of NAD^+^ to NADH, resulting in an increase in absorbance at 340 nm [13,14]. The optimization of the enzyme-coupled assay and steady-state kinetic analysis of PatA acetyltransferases were performed as described previously [12].

### Western blotting assays and peptide fingerprinting using MS

Protein samples were electrophoresed on an SDS/PAGE and then transferred on to a PVDF membrane for 1 h at 100 V. The membrane was blocked in BSA blocking buffer for 2 h at 25°C. We used an acetyl-lysine antibody (Cat# ICP0381, ImmuneChem Pharmaceuticals Inc., Canada) diluted to 1:15000. After incubation at 4°C for 10–12 h, the blot was washed with TBS and Tween 20 (TBST) three-times at ambient temperature. Chemiluminescence was detected using the ECL enhanced system and a luminescent image analyzer (DNR Bio Imaging Systems, Israel). To continuously monitor the acetyl-lysine sites, the acetylated Acs were isolated using the SDS/PAGE and proteins were solubilized according to previously described filter-aided sample preparation (FASP) procedures [15]. Predigested proteins were extracted by solid-phase extraction, separated using Nano-HPLC, and analyzed on an Orbitrap Fusion™ Tribrid™ Mass Spectrometer (Thermo Scientific, U.S.A.). Tandem MS (MS/MS) spectra were searched using the MASCOT engine (version 2.2; Matrix Science, United Kingdom).

### *In vitro* assays of Acs

The specific activity of Acs was determined at 30°C in a transparent 384-well microplate reader (Bio Tek Instruments, Winooski, U.S.A.) at 340 nm [16]. The standard reaction mixture contained 100 mM Tris/HCl (pH 7.8), 10 mM malate (pH 7.7), 0.2 mM CoA, 8 mM ATP (pH 7.5), 1 mM NAD^+^, 10 mM MgCl_2_, 3 units of malate dehydrogenase, 0.4 units of citrate synthase, and 0.3 μM *Sve*Acs or *Ami*Acs. The reaction was initiated with 100 mM potassium acetate and the rate was determined continuously for 5 min by measuring NADH production at 340 nm. One unit was defined as the conversion of 1.0 μmol of NAD^+^ to NADH per min in the presence of saturating levels of acetate.

### CD spectroscopy

CD spectra were prepared using a CD spectrophotometer (Applied Photophysics, U.K.) and 1 mm cuvette equilibrated at 25°C. All samples were desalted and dialyzed against buffer P (150 mM KF, 94 mM K_2_HPO_4_, and 6 mM KH_2_PO_4_, pH 8.0). Samples were diluted to 0.25 μg/μl and incubated with and without 2 mM Asn or 2 mM Cys. The CD spectra were collected between 190 and 260 nm, and three repeat scans were averaged. Parameters of the secondary structure were analyzed using CDNN 2.1 (http://bioinformatik.biochemtech.uni-halle.de/cdnn).

### Site-directed mutagenesis and purification of PatA and Acs

Mutations at key sites in PatA and Acs proteins were introduced into pET28a using QuikChange Mutagenesis Kit (TransGen, Beijing, China) according to previously described procedure [12,17]. The mutations were confirmed by DNA sequencing. The overexpression and purification of mutant proteins were performed as described above.

### Structural model of the ACT domain of *Ami*PatA

The structure of the ACT domain of *Ami*PatA was built by homology modeling from Swiss Model server (https://swissmodel.expasy.org/) using four ACT domains with the greatest similarity to that of *Ami*PatA (PDB ID: 3W7B, 3O1L, 1PSD, 2J0X) according to the previously described procedure [12]. Discovery Studio 3.5 Client was used as the docking tool to predict amino acid-binding to AAPatA proteins. CHARMM forcefield was used to optimize the model of the ACT domain, and CFF forcefield was used to optimize the structure of Cys. We added Kollman charge to the residues of ACT domain of *Ami*PatA. LibDock was used to perform molecular docking. The conserved binding site was selected as docking center, and a sphere with a radius of 10 Å were considered as binding region for docking studies.

### Sequences and phylogenetic tree analysis of ACT and GNAT domains

Multiple alignments of the ACT and GNAT domains, as well as a phylogenetic analysis of full-length sequences of all ACT-GNAT proteins, were performed using ClustalW 2.1 and MEGA 5.10 software.

### Data and statistical analysis

Microsoft Excel was used to quantitate protein concentrations and the activities of PatA, *Sve*Acs, and *Ami*Acs. GraphPad Prism 5.0 was used for statistical analyses, preparation of graphs, and data analysis. Data are presented as means ± S.E.M. for three independent experiments (*n*=3); statistical significance was accepted at *P*≤0.05.

## Results

### PatA acetyltransferases with ACT-GNAT domain organization are found in actinobacteria

**Figure 1:**
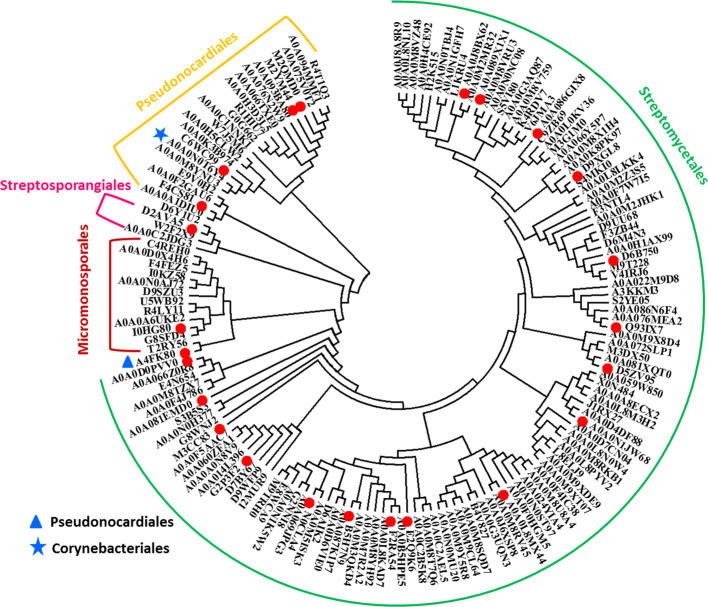
Phylogenetic tree analysis of AAPatA sequences The phylogenetic tree was made using EMGA 5.10. All ACT-GNAT protein sequences in the InterPro database (v.54) were conducted using ClustalW 2.1 and then construct ed the neighbor tree. The parameters used for the analysis was default value. The specific parameters were as follows: gap opening penalty of pairwise alignment 10, gap extension penalty of pairwise alignment 0.1, gap opening penalty of multiple alignment 10, gap extension penalty of multiple alignment 0.2, gap separation dstance 4, delay divergent cutoff 30%.

Previous work has shown that *Ma*Kat (Micau_1670, from *M. aurantiaca*) containing an ACT-GNAT domain organization is an amino acid-regulated Pat that can acetylate Ac–CoA synthetase [12]. One hundred and fifty-six proteins containing the ACT-GNAT domain organization were displayed in the InterPro database (v.54.0) and the sequences of these proteins were examined. Six repeated sequences were deleted. A total of 150 unique ACT-GNAT acetyltransferases, which we designated as AAPatAs, are listed in Supplementary Table S1. The AAPatA enzymes are unique to and are distributed in 22 species of actinobacteria. Remarkably, 107 AAPatAs (71.3%) were found in *Streptomyces*. Some AAPatAs were found in *Amycolatopsis* (10), *Actinoplanes* (5), and *Micromonospora* (5). We performed a phylogenetic tree analysis using the protein sequences of all AAPatAs (Figure 1). Figure 1 shows that the sequence of AAPatA reflects the phylogeny of actinobacteria. In AAPatA sequence phylogeny, organisms are clustered according to orders, such as Streptomycetales (*Streptomyces, Kitasatospora*), Micromonosporales (*Actinoplanes, Micromonospora, Verrucosispora*), Pseudonocardiales (*Saccharopolyspora, Pseudonocardia, Saccharothrix, Actinosynnema, Kibdelosporangium, Alloactinosynnema, Actinokineospora, Prauserella, Amycolatopsis*), and Streptosporangiales (*Streptomonospora, Microbispora, Streptosporangium*). Exceptions included A4FK80 from *Saccharopolyspora erythraea* DSM 40517 (Pseudonocardiaceae), which was between Streptomycetales and Micromonosporales, and A0A0N0T6Y4 from *Nocardia* sp. NRRL S-836 (Corynebacteriales), which was dispersed amongst the Pseudonocardiales. The results revealed the sequence evolution of AAPatA acetyltransferases in actinobacteria, which can be used as a molecular marker for speciation of actinobacteria.

### AAPatA can be divided into two groups: Cys- and Asn-dependent Pat

We overexpressed and purified 24 AAPatA acetyltransferases (indicated by the red dot in Figure 1) from all the ACT-GNAT proteins (Table 1). Like *Ma*Kat from *M. aurantiaca* and other Pat (*Sl*Pat, *Af*Pat, 1YGH_A: yeast, 1Z4R_A: human, 1QSN_A: TeT), the most highly conserved motif QXXGX (G/A) for Ac–CoA recognition and binding, and a glutamic acid residue provides as an active site to deprotonate the lysine residue of the acetyl acceptor, were observed in GNAT domains of all 24 AAPatAs (Supplementary Figure S1). The experimental data showed that all AAPatAs can directly acetylate the putative AMP-forming Ac–CoA synthetase (*Ami*Acs from *Actinosynnema mirum* strain DSM 43827) *in vitro* (Supplementary Figure S2). These results demonstrate that AAPatA is a novel type of Pat.

**Table 1:** Amino acid ligands of 24 AAPatA proteins identified in the present study

No.	Names	UniProt accession	Species	Ligands
1	*Svr*PatA	A0A0J8BX62	*Streptomyces viridochromogenes*	Asn
2	*Sgu*PatA	A0A089X1X1	*Streptomyces glaucescens*	Asn
3	*Scb*PatA	A0A086GIX8	*Streptomyces scabiei*	Asn
4	*Svt*PatA	D9XGL8	*Streptomyces viridochromogenes* DSM 40736	Asn
5	*Sal*PatA	D6B750	*Streptomyces albus* J1074	Asn
6	*Sco*PatA	Q93IX7	*Streptomyces coelicolor* ATCC BAA-471	Asn
7	*Sgh*PatA	D5ZV95	*Streptomyces ghanaensis* ATCC 14672	Asn
8	*Sch*PatA	A0A0N1JW68	*Streptomyces chattanoogensis*	Asn
9	*Svg*PatA	A0A0L8MX44	*Streptomyces virginiae*	Asn
10	*Scl*PatA	E2Q9K6	*Streptomyces clavuligerus* DSM 738	Asn
11	*Sve*PatA	F2RA54	*Streptomyces venezuelae* DSM 40230	Asn
12	*Spr*PatA	B5H7N9	*Streptomyces pristinaespiralis* ATCC 25486	Asn
13	*Sfi*PatA	N0CLA4	*Streptomyces fulvissimus* DSM 40593	Asn
14	*Svl*PatA	G2P3F2	*Streptomyces violaceusniger* Tu 4113	Asn
15	*Smb*PatA	M3CC83	*Streptomyces mobaraensis* DSM 40847	Asn
16	*Sfr*PatA	A0A081EMD0	*Streptomyces fradiae*	Asn
17	*Kgr*PatA	A0A0D0PVV0	*Kitasatospora griseola*	Asn
18	*Sen*PatA	A4FK80	*Saccharopolyspora erythraea* DSM 40517	Cys
19	*Ams*PatA	I0HG80	*Actinoplanes missouriensis* DSM 43046	Cys
20	*Sro*PatA	D2AVA5	*Streptosporangium roseum* DSM 43021	Cys
21	*Pdx*PatA	F4CS81	*Pseudonocardia dioxanivorans* DSM 44775	Cys
22	*Ami*PatA	C6WC99	*Actinosynnema mirum* DSM 43827	Cys
23	*Ade*PatA	M2YM44	*Amycolatopsis decaplanina* DSM 44594	Cys
24	*Aaz*PatA	M2QMD0	*Amycolatopsis azurea* DSM 43854	Cys

**Figure 2:**
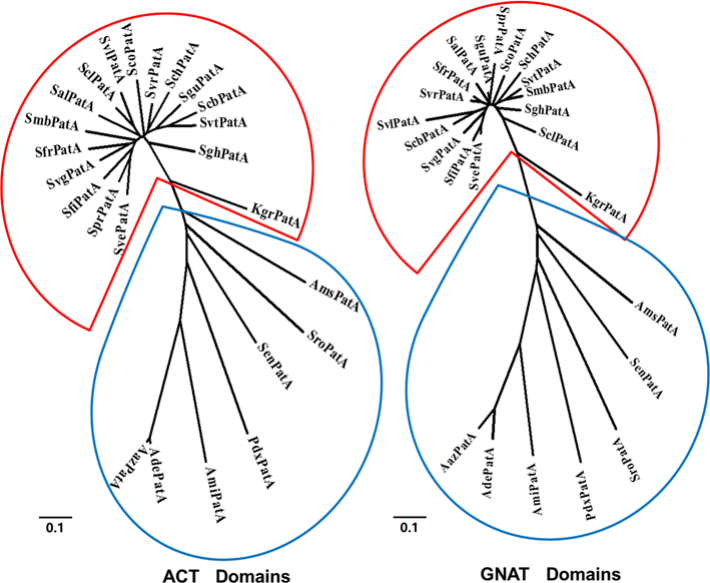
Phylogenetic analysis of the GNAT and ACT domain sequences in AAPatA proteins The GNAT and ACT domains of the 24 AAPatA proteins were analyzed using a phylogenetic tree analysis. The two groups were clustered according to the amino acids Asn (indicated by the red lines) and Cys (indicated by the blue lines) in ACT and GNAT domains. The phylogenetic tree was made using EMGA 5.10. All the GNAT or ACT domain sequences were conducted using ClustalW 2.1 and then constructed the neighbor tree. The parameters used for the analysis was default value. The specific parameters were as follows: gap opening penalty of pairwise alignment 10, gap extension penalty of pairwise alignment 0.1, gap opening penalty of multiple alignment 10, gap extension penalty of multiple alignment 0.2, gap separation dstance 4, delay divergent cutoff 30%.

The ACT domain binds to more than one amino acid, by inducing conformational changes, it can generate variation in the activity of the catalytic domain. To identify the amino acid ligands of ACT domains in AAPatA acetyltransferases, we investigated the activities of all AAPatAs acetylating putative Ac–CoA synthetases with and without the addition of the 20 L-amino acids. Acetylation was measured continuously using a coupled enzymatic assay to monitor increase in absorbance at 340 nm (ΔA_340_). These increases resulted from the formation of NADH by pyruvate dehydrogenase because of AAPatA-dependent CoA production [12–14]. The ACT domain allosterically regulates acetylation activity of AAPatA in response to addition of amino acids, as shown in Supplementary Figure S2. Adding Cys or Asn increased the values of ΔA_340_, while no change in the values of ΔA_340_ were observed after the addition of other amino acids. These increases in the values of ΔA_340_ suggest that the acetylation activity of AAPatAs is enhanced by the conformational changes resulting from Cys or Asn binding the ACT domain. The amino acid ligands of AAPatAs are listed in Table 1. We found that members of the AAPatA family are divided into two groups based on the allosteric ligands of the ACT domain: the Asn-regulated PatAs and Cys-regulated PatAs. The former are mainly found in *Streptomyces*; the latter are distributed in other actinobacteria. Furthermore, we performed a phylogenetic analysis of the sequences that make up the GNAT and ACT domains in the AAPatA proteins. As shown in Figure 2, the two groups were clustered according to the binding ligands, Asn (indicated by the red lines) or Cys (indicated by the blue lines) in both ACT and GNAT domains.

**Figure 3:**
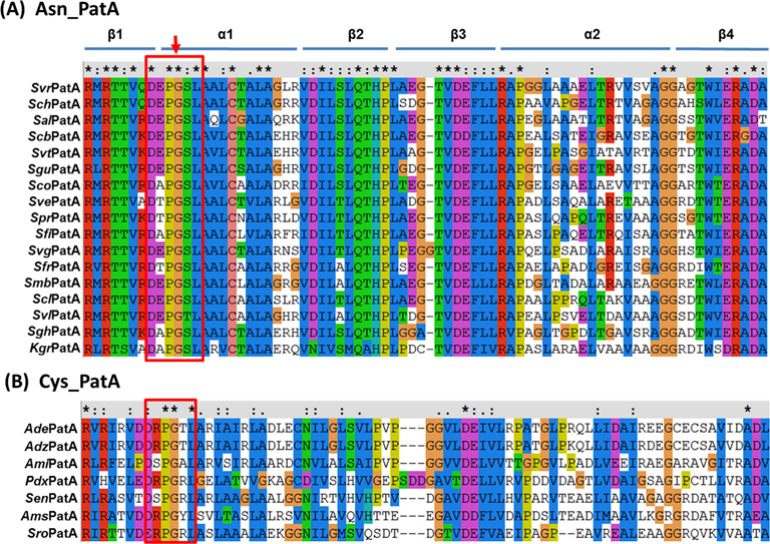
Multiple alignment of ACT domains (**A**) An alignment of amino acid sequences that make up the ACT domains of 17 proteins regulated by Asn. (**B**) Multiple alignment of amino acid sequences that make up the seven ACT domains of AAPatA regulated by Cys. The red frame shows important region between the first β1 strand and the first α1 helix containing a highly conserved glycine residue.

Multiple alignments of ACT domains in the 24 PatA proteins revealed that all ACT domains contain the conserved βαββαβ-fold and the highly conserved glycine residue between the first β1 strand and the first α1 helix; Asn-binding ACT domains have higher sequence conservation than the Cys-binding ACT domains (Figure 3). The β1 and α1 are the conserved motifs for amino acid recognition and binding [18]. As indicated by the red frame in Figure 3, conserved residues, such as DXP**G**XL, at the interface of β1 and α1, may play an important role in the binding of specific amino acids. Bioinformatics analysis and coupled enzymatic assay showed that AAPatAs, the protein lysine acetyltransferases (KATs) that acetylate the Ac–CoA synthetase, are divided into two groups: Cys_AAPatA and Asn_PatA. Next, we characterized *Ami*PatA (Cys_AAPatA) from *A. mirum* DSM 43827 and *Sve*PatA (Asn_PatA) from *S. venezuelae* DSM 40230.

### Cys enhances protein lysine acetylation by *Ami*PatA derived from *A. mirum*

**Figure 4:**
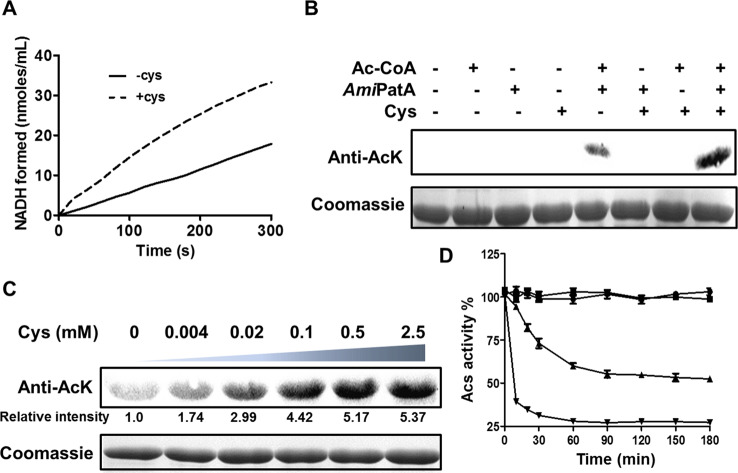
Cys-mediated allosteric regulation of *Ami*PatA activity for protein acetylation (**A**) Acetyltransferase activity of *Ami*PatA was measured using a coupled enzymatic assay with and without Cys. (**B**) Acetylation of *Ami*Acs by *Ami*PatA. *Ami*Acs (2 μM) was incubated alone or in the presence of *Ami*PatA (0.3 μM), Ac–CoA (60 μM), or Cys (2 mM) in a volume of 100 μl at 37°C for 1.5 h, followed by analysis using SDS/PAGE. Levels of acetylation were detected by Western blot assay using an anti-AcK antibody. (**C**) Acetylation levels of *Ami*Acs were measured using Western blot assay. Varying concentrations of Cys, ranging from 0 μM to 2.5 mM, were added to the solution of *Ami*Acs (2 μM), *Ami*PatA (0.3 μM), and Ac–CoA (60 μM), followed by incubation for 1.5 h at 37°C. (**D**) Time-dependent inactivation of *Ami*Acs by acetylation. The activity of *Ami*Acs was measured at different time intervals during incubation with *Ami*PatA. ● 5 μM *Ami*Acs; ▪ 5 μM *Ami*Acs and 0.2 μM *Ami*PatA; ▲ 5 μM *Ami*Acs, 0.2 μM *Ami*PatA, and 60 μM Ac–CoA ▼5 μM *Ami*Acs, 60 μM Ac–CoA, 0.2 μM *Ami*PatA and 2 mM Cys. Each data point indicate an average of three independent assays; mean ± S.E.M. (*P*<0.05; *n*=3 experiments).

To examine whether Cys regulates the enzymatic activity of *A. mirum*-derived *Ami*PatA, we used a spectrophotometric assay to assess the acetylation of *Ami*Acs by *Ami*PatA in the presence and absence of Cys. As shown in Figure 4A, the acetylation of Acs was enhanced in the presence of Cys, indicating an increase in the activity of the acetyltransferase *Ami*Acs. The initial rate of acetylation of *Ami*Acs by *Ami*PatA (indicated by the formation of NADH) was estimated to be 3.8 μM/min, which increased to 6.8 μM/min (an increase by 179%) in the presence of Cys. The acetylation level of *Ami*Acs was assessed by Western blotting using an anti-acetyl-lysine antibody (Anti-AcK) in the presence or absence of Cys. Cys increased the acetylation level of *Ami*Acs (Figure 4B); furthermore, increases in the acetylation level of *Ami*Acs were dependent on the concentration of Cys (Figure 4C). When the concentration of Cys was increased from 0.004 to 0.5 mM, the acetylation levels also increased. The concentrations of Cys ligand that saturated the acetylation of the protein were in the submillimolar range. The concentration is within the endogenous ranges reported for Cys, which are estimated to be 0.1–0.2 mM in *E. coli* cells [19], indicating that intracellular Cys is able to exert an effect on activity of *Ami*PatA enzyme *in vivo*. To investigate the effect of acetylation on enzyme activity, *Ami*Acs was incubated with *Ami*PatA in the presence or absence of Ac–CoA for 3 h. The activity of *Ami*Acs was reduced by half in the presence of Ac–CoA and *Ami*PatA, indicating that lysine acetylation effectively decreased the activity of *Ami*Acs (Figure 4D). The acetylated *Ami*Acs protein was subjected to trypsin digestion, and the generated peptides were analyzed by MS. The acetylated lysine site (SG**K** (620) IMR) was identified in the conserved PXXXXGK motif of the AMP-forming acyl–CoA synthetases; this motif is recognized by the previously reported bacterial acetyltransferases [17] (Supplementary Figure S3). We further determined the kinetic parameters of the *Ami*PatA catalyzed acetylation reaction of *Ami*Acs in the absence and presence of Cys using a coupled enzymatic assay previously described [12]. The data are shown in Table 2. The *K*_m_ and *k*_cat_ values of *Ami*PatA for *Ami*Acs are 45.5 μM and 0.03 s^−1^, respectively. Cys was able to decrease the *K*_m_ value of *Ami*PatA for *Ami*Acs by three-fold, and increase *k*_cat_ value by 1.5-fold.

**Table 2:** Kinetic analysis of *Ami*PatA on *Ami*Acs

Enzyme	Substrate	K_m_/μM	*k*_cat_/s^−1^	*k*_cat_/*K*_m_ (M^−1^s^−1^)
***Ami*PatA**	Ac–CoA	5.5 ± 1.1	0.19 ± 0.008	(3.6 ± 0.8) × 10^4^
	*Ami*Acs	45.5 ± 7.3	0.30 ± 0.014	(6.8 ± 1.4) × 10^3^
***Ami*PatA (Cys)**	Ac–CoA	4.2 ± 0.6	0.33 ± 0.010	(8.2 ± 0.7) × 10^4^
	*Ami*Acs	15.9 ± 1.8	0.54 ± 0.014	(3.4 ± 0.5) × 10^4^

**Figure 5:**
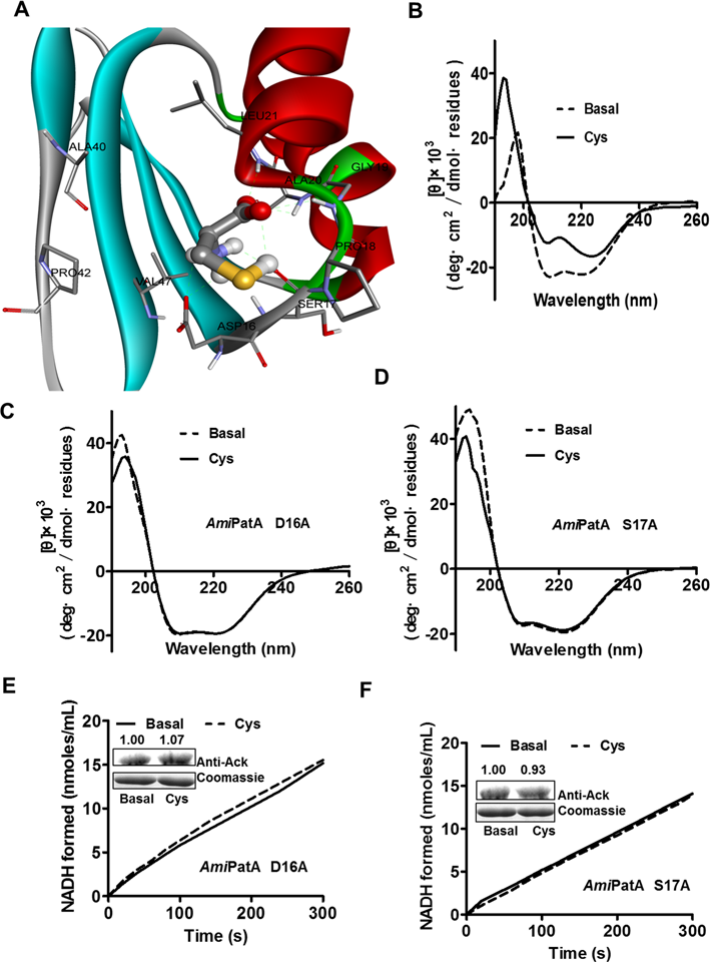
Specific binding residues of Cys in the ACT domain of *Ami*PatA (**A**) A structural model of the ACT domain of *Ami*PatA shows that the possible residues of *Ami*PatA interact with Cys via hydrogen bonding between the first strand (β1) and the first helix (α1). (**B**) Secondary structure (dotted line) and conformational change of *Ami*PatA, after the addition of Cys (full line) and subtraction of buffer baseline, are shown from 190 to 260 nm. (**C**,** D**) The conformational changes in *Ami*PatA mutants (D16A and S17A) were evaluated using CD in the presence and absence of Cys. (**E**,** F**) Initial rate of the formation of NADH and Western blotting analysis are shown in the *Ami*PatA mutants D16A and S17A. The gray values were quantitated by densitometry using ImageJ software.

The ACT domain is an amino acid-binding domain involved in amino acid metabolism; 3-phosphoglycerate dehydrogenase (3PGDH) in *E. coli* was the first enzyme found to contain the ACT domain [20] and folds with a ferredoxin-like topology. In the ACT domain, the most conserved part is the region at the interface between the first strand (β1) and the first helix (α1); the residues binding specific amino acids via hydrogen bonding are found at this conserved region [21]. To investigate key residues used to bind Cys in *Ami*PatA, the structure of the ACT domain in *Ami*PatA was modeled using four ACT domains with the greatest similarity (query cover: 85%, identity: 25%) to that of *Ami*PatA (PDB ID: 3W7B, 3O1L, 1PSD, 2J0X). The docking result showed that the residues involved in hydrogen bonding with Cys were Asp^16^, Ser^17^, Gly^19^, Ala^20^, and Leu^21^; in particular, Asp^16^ and Ser^17^ were located in the loop between the first strand (β1) and the first helix (α1) of the *Ami*PatA ACT domain (Figure 5A). The characteristic Gly^19^, followed by a hydrophobic residue, is the most conserved residue in the helix, necessary for maintaining the conformation of the strand–helix interface. Far-UV CD spectroscopy was conducted to confirm the conformational change in *Ami*PatA after the addition of Cys (Figure 5B). CD spectra, recorded from 190 to 260 nm, showed an increase in ellipticity at 208 and 222 nm, indicating a decrease in the α-helical content of *Ami*PatA after the addition of Cys and suggesting that the binding of Cys altered the secondary structure of the protein. The characteristics of the secondary structure, calculated using CDNN version 2.1, indicated the presence of approximately 45.1% α-helices and 11.1% β-sheets in *Ami*PatA, and 35.8% α-helices and 15.8% β-sheets in *Ami*PatA after the addition of Cys. This phenomenon was observed in some proteins regulated allosterically by effectors [22,23].

To further identify the key residues involved in the binding of Cys, we mutated the acidic amino acid Asp^16^ and the hydrophilic amino acid Ser^17^ to a neutral and hydrophobic amino acid alanine. The CD spectra of D16A and S17A mutant proteins showed that Cys does not induce changes in the secondary structures of *Ami*PatA mutants (Figure 5C,D). We also used an enzyme-coupled assay and Western blotting analysis to investigate the acetylation activity of the two mutants with and without the addition of Cys. As shown in Figure 5E,F, no increases in acetylation were observed in D16A and S17A mutants after the addition of Cys; this agrees with the CD assays that indicated no conformational changes induced by the addition of Cys. These results suggest that Asp^16^ and Ser^17^ at the interface between β1 and α1 may somehow affect the Cys-binding of *Ami*PatA. Taken together, these results indicate that *Ami*PatA has protein-acetylating acetyltransferase activity and that this activity is regulated allosterically by Cys binding.

### Asn enhances protein lysine acetylation by *Sve*PatA from *S. venezuelae*

**Figure 6:**
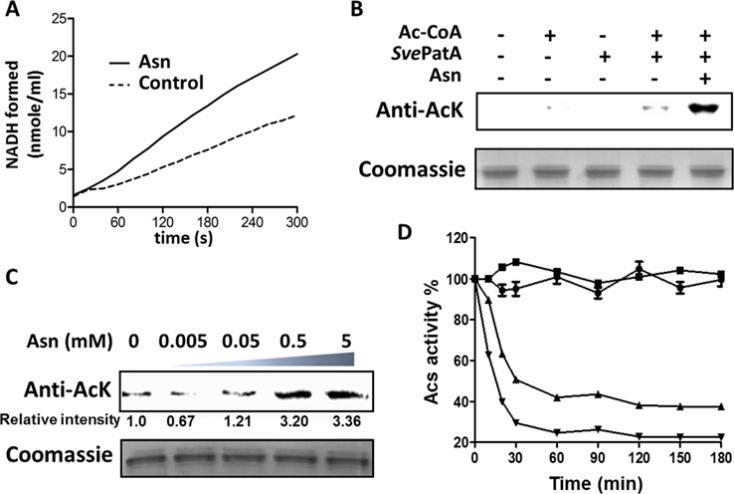
Asn-mediated allosteric regulation of *Sve*PatA activity for protein acetylation (**A**) Acetyltransferase activity of *Sve*PatA was measured using a coupled enzymatic assay with and without the addition of Asn. (**B**) Acetylation of *Sve*Acs by *Sve*PatA. *Sve*Acs (2 μM) was incubated alone or in the presence of *Sve*PatA (0.3 μM), Ac–CoA (60 μM), and Asn (2 mM) in a volume of 100 μl at 37°C for 1.5 h, followed by an analysis using SDS/PAGE. Levels of *Sve*Acs acetylation were detected by Western blotting using a specific anti-AcK antibody. **(C)** Levels of *Sve*Acs acetylation were measured using a Western blot assay. Varying concentrations of Asn, in the range from 0 μM to 5 mM, were added to a solution containing *Sve*Acs (2 μM), *Sve*PatA (0.3 μM), and Ac-CoA (60 μM), followed by incubation for 1.5 h at 37°C. (**D**) Time-dependent inactivation of *Sve*Acs by acetylation. The activity of *Sve*Acs was measured at different time intervals during incubation with *Sve*PatA. ● 5 μM *Sve*Acs; ▪ 5 μM *Sve*Acs and 0.2 μM *Sve*PatA; ▲ 5 μM *Sve*Acs, 0.2 μM *Sve*PatA, and 60 μM Ac-CoA ▼5 μM *Sve*Acs, 60 μM Ac-CoA, 0.2 μM *Sve*PatA, and 2 mM Asn. Each data point indicate the average from three independent assays. Data are represented as mean ± S.E.M., *n*=3 experiments; the significance level was set at *P*<0.05.

Finally, we characterized *Sve*PatA from *S. venezuelae* as a model representative of Asn-binding AAPatA acetyltransferases. To investigate the effect of Asn on the activity of *Sve*PatA, we determined the initial rate of *Sve*Acs acetylation by *Sve*PatA with and without the addition of Asn. For this, we used a coupled enzymatic assay, where the amount of CoA released after *Sve*Acs acetylation was measured by the formation of NADH from NAD^+^ via pyruvate dehydrogenase. As shown in Figure 6A, *Sve*PatA acetylated *Sve*Acs in the absence of Asn (2.23 nmol of NADH formed per min/ml), and the rate of acetylation was increased by 176% in the presence of Asn (3.92 nmol of NADH formed per min/ml). Western blotting was conducted to detect the level of *Sve*Acs acetylation with an anti-Ack antibody in the presence and absence of Asn. Acetylation of *Sve*Acs was clearly observed after incubation with *Sve*PatA and Ac–CoA; acetylation was significantly increased in the presence of Asn (Figure 6B). Moreover, the increase in the acetyltransferase activity of *Sve*PatA was dependent on the concentration of Asn (Figure 6C). Previous work showed that Asn can reach cytoplasmic concentration of 0.51 mM in *E. coli* cells [24], which is comparable with the concentration of Asn needed to regulate allosterically activity of *Sve*PatA enzyme. To examine the effect of acetylation on enzyme activity, *Sve*Acs was incubated with *Sve*PatA in the presence of Ac-CoA for 2 h. The activity of *Sve*Acs was reduced by approximately 60%, indicating that lysine acetylation effectively decreased the activity of *Sve*Acs (Figure 6D). These results demonstrated that AAPatA proteins possessing the ACT-GNAT domain in non-Streptomycetaceae actinobacteria can function as acetyltransferases regulated allosterically by Asn.

**Figure 7:**
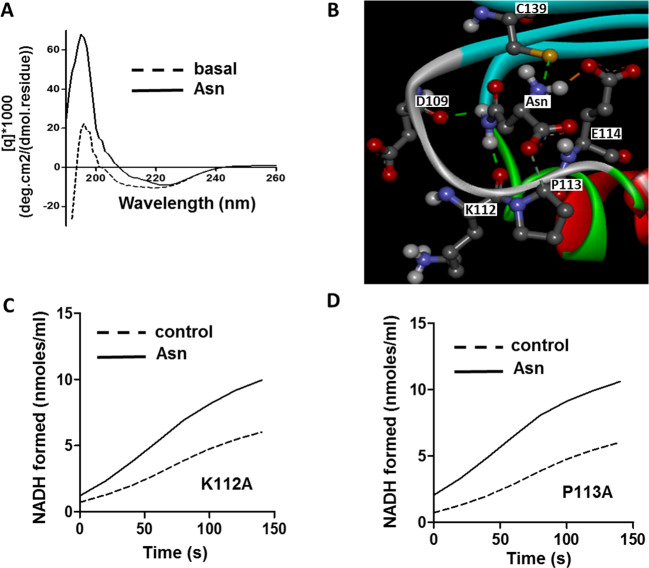
The binding of Asn with *Sve*PatA (**A**) Secondary structure (dotted line) and conformational change of *Sve*PatA, after the addition of 2 mM Asn (full line) and subtraction of buffer baseline, are shown from 190 to 260 nm. (**B**) The docking result of Asn to *Sve*PatA shows that the possible residues of *Sve*PatA interact with Asn via hydrogen bonds. (**C**,** D**) The acetyltransferase activities of *Sve*PatA mutants (K112A and P113A) in the presence of Asn.

As shown in Figure 7A, CD spectra of *Sve*PatA displayed an increase in ellipticity at 208 and 222 nm after the addition of Asn, indicating that the binding of Asn altered the secondary structure of the protein. The docking result of Asn to *Sve*PatA showed that the residues involved in hydrogen bonding with Asn were Asp^109^, Lys^112^, Pro^113^, Glu^114^, and Cys^139^ (Figure 7B). To further identify the key residues involved in the binding of Asn, we mutated the acidic amino acid Lys^112^ and amino acid Pro^113^ to alanine. Allosteric effect of Asn on *Sve*PatA activity was still observed in two mutants K112A and P113A (Figure 7C,D), indicating that Lys^112^ and Pro^113^ were not involved in the Asn binding of *Sve*PatA.

## Discussion

*N*-lysine acetylation is a dynamic, reversible, and regulatory post-translational modification (PTM) used to modulate enzyme activity in prokaryotes. Accumulating evidence suggests that metabolic networks are co-ordinated via reversible acetylation of enzymes that regulate cellular metabolism to maintain homeostasis in the rapidly changing microenvironment. The present study examined the archetypical acetyltransferases AAPatAs possessing GNAT and ACT domains, and shows a novel signaling pathway for regulating the acetylation of cellular proteins. These findings indicate that in actinobacteria, acetyltransferase activity and protein acetylation may be tightly correlated with intracellular amino acid metabolism.

The conventional mechanism for reversible *N*^ε^-acetylation of proteins is enzyme-catalyzed acetylation and deacetylation, which relies on KATs and deacetylases (HDACs). Reversible enzyme-catalyzed acetylation is regulated at different levels, such as transcriptionally and allosterically, to adjust the acetylation of specific proteins responding to intracellular nutritional signals. In *B. subtili*s, the *acuA* gene encoding an acetyltransferase is controlled by the carbon catabolite control protein, CcpA [5]. In *E. coli*, the cAMP receptor protein CRP induces the expression of *yfiQ*, which encodes the acetyltransferase YfiQ, thus increasing the acetylation level of proteins in response to the intracellular cAMP signal [6]. In *S. enterica*, lolR, the repressor of *myo*-inositol catabolism, activates the transcription of *pat* and c*obB* [10].

There are three classes of KATs that catalyze the donation of an acetyl group from acetyl-coenzyme A (Ac-CoA) to an ε-amino group of a deprotonated lysine. These KATs include the GNAT family, the MYST family, and the p300/CBP family [25]. The genomes of actinobacteria encode approximately 40–80 GNAT acetyltransferases, 1–2 NAD^+^-dependent sirtuin deacetylases, or 1 NAD^+^-independent protein deacetylase. The genome of *S. coelicolor* encodes 77 putative GNAT acetyltransferases (Pfam00583), 2 sirtuin-type deacetylases (SCO0452, SCO6464), and an AcuC-like NAD^+^-independent deacetylase (SCO3330). The genome of *S. avermitilis* encodes 43 putative GNAT acetyltransferases, 1 sirtuin-type deacetylase (SAV_537), and 1 AcuC-like deacetylase (SAV_4729). The genome of *S. lividans* encodes 72 putative GNAT acetyltransferases, 2 sirtuin-type protein deacetylases (EFD65580, EFD71509), and 1 NAD^+^-independent deacetylase (EFD68590). The genome of *Saccharopolyspora erythraea* encodes 42 putative GNAT acetyltransferases, 1 NAD^+^-dependent deacetylase (SACE_3798), and 1 NAD^+^-independent deacetylase (SACE_1779). Thus, the number of GNAT acetyltransferases in a bacterial species may reflect the metabolic complexity of the species. Thus far, all the known bacterial Pat identified contain the GNAT domain having low sequence homology, and sharing a conserved motif A, sequence (R/Q)XXGX(G/A) for Ac–CoA recognition and binding, and a glutamate residue providing as an active site. It is worth noting that there has been considerable debate about the function of the conserved glutamate as a catalytic base [26,27]. In prokaryotes, some Pat have two different domain organizations: the multidomain (Class I) and single-domain (Class II). Several single-domain GNAT proteins have been shown to have acetyltransferase activity. These Class II enzymes, which are approximately 200 residues long, include *Bs*AcuA from *B. subtilis* [28], *Rp*KatA from *Rhodopseudomonas palustris* [29], and *Sac*AcuA from *Saccharopolyspora erythraea* [17].

Class Iα Pat, such as the first discovered *Se*Pat in *S. enterica* [1], the homologs (*Ec*PatZ from *E. coli* and *Rp*Pat from *R. palustris*) and variants (*Sl*PatA from *Streptomyces lividans*) of *Se*Pat [6,29,30], are 800-1000 residues long and contain a GNAT catalytic and NAD^+^-forming CoA ligase domains; the function of the latter is undetermined, but it has homology with ADP-forming acyl–CoA synthetases. The catalytic mechanism and domains function of *Se*Pat and *Ec*PatZ have been investigated [31,32]. Biochemical and thermodynamic analyses of *Se*Pat revealed that *Se*Pat has two distinct sites for Ac–CoA binding, and shows the positive co-operativity (with a Hill coefficient of 2.2 ± 0.2) where binding of Ac–CoA to one site increases the affinity of a second binding site [31]. Recent work found that the kinetic data of *Ec*PatZ for Ac–CoA display atypical sigmoidal activity, suggesting that *Ec*PatZ also reveals the positive co-operativity with a Hill coefficient of 7.91 ± 0.22 [32]. Positive co-operativity observed in class Iα multidomain acetyltransferases may be a result of subunit interactions and oligomerization in the presence of Ac–CoA. More recently, an interesting work demonstrated that cAMP directly binds to *Se*Acs, and inhibits its activity through competitive binding to the ATP/AMP pocket, whose binding with cAMP promotes *Se*Acs acetylation, and further resulting in inhibition of *Se*Acs activity [33].

Class Iβ Pat are allosteric enzymes with two domains in which an effector-binding regulatory domain is fused to the GNAT catalytic domain. The cAMP-sensing acetyltransferases (*Mt*PatA in *Mycobacterium tuberculosis* and *Ms*PatA in *M. smegmatis*) [34,35], NADP-sensing acetyltransferase (*Mx*Kat in *Myxococcus xanthus*) [36], and amino acid-sensing acetyltransferase (*Ma*Kat in *Micromonospora aurantiaca*) [12] are examples of Class Iβ Pat. These enzymes have regulatory domains that bind to an effector (e.g. cAMP, NADP, or amino acids) to allosterically regulate the activities of Pat; this presents a novel mechanism for directly connecting the levels of protein acetylation with intracellular concentration of metabolites. Class Iβ enzymes are not widely distributed in microorganisms. cAMP-sensing acetyltransferases are present only in mycobacteria, while the NADP-sensing acetyltransferase is present only in *M. xanthus*. In the present study, we found that the amino acid-sensing AAPatA acetyltransferases are divided into two groups based on allosteric effectors: the Asn-regulated PatA and Cys-regulated PatA. The former are found only in Streptomycetaceae; the latter are distributed in other actinobacteria (Pseudonocardiaceae, Micromonosporaceae, Nocardiopsaceae, and Streptosporangiaceae). Class Iβ Pat with different regulatory domains use diverse metabolic signals to regulate lysine acetylation in response to varying physiological conditions and environmental changes. To investigate the function of Class Iβ enzymes, it is necessary to identify their physiological substrates. A universal stress protein USP (MSMEG_4207), Acs, and eight additional acyl-CoA synthetases may be the possible acetylated protein targets of *Ms*PatA, and their acetylation may be dependent upon the levels of intracellular cAMP [11,12,34]. No acetylated protein targets of other Class Iβ enzymes have been identified. It is possible that AAPatA may acetylate enzymes involved in Asn and Cys metabolism and may regulate these pathways in response to intracellular Asn or Cys concentrations. More work is needed to identify the physiological substrates of Class Iβ enzymes and to elucidate the effect of acetylation on the regulation of metabolic enzyme activity.

## Supplementary Material

Supplementary Figure S1-S3 and Table S1

## Abbreviations

AAPatA: amino acid sensing acetyltransferase
Ac–CoA: acetyl–CoA
AcP: acetyl-phosphate
Acs: acetyl–CoA synthetase
anti-Ack: anti-acetyl-lysine antibody
CcpA: catabolite control protein A
GNAT: Gcn5-related *N*-acetyltransferase
KAT: lysine acetyltransferase
Pat: protein acetyltransferase

## References

[1] Starai V.J. and Escalante-Semerena J.C. (2004) Identification of the protein acetyltransferase (Pat) enzyme that acetylates acetyl-CoA synthetase in *Salmonella enterica*. J. Mol. Biol. 340, 1005–1012 15236963 10.1016/j.jmb.2004.05.010

[2] Starai V.J., Celic I., Cole R.N., Boeke J.D. and Escalante-Semerena J.C. (2002) Sir2-dependent activation of acetyl-CoA synthetase by deacetylation of active lysine. Science 298, 2390–2392 12493915 10.1126/science.1077650

[3] Weinert B.T., Iesmantavicius V., Wagner S.A., Schölz C., Gummesson B., Beli P. et al. (2013) Acetyl-phosphate is a critical determinant of lysine acetylation in *E. coli*. Mol. Cell 51, 265–272 23830618 10.1016/j.molcel.2013.06.003

[4] Kuhn M.L., Zemaitaitis B., Hu L., Sahu A., Sorensen D., Minasov G. et al. (2014) Structural, kinetic and proteomic characterization of acetyl phosphate-dependent bacterial protein acetylation. PLoS ONE 9, e94816 24756028 10.1371/journal.pone.0094816 PMC3995681

[5] Grundy F.J., Turinsky A.J. and Henkin T.M. (1994) Catabolite regulation of *Bacillus subtilis* acetate and acetoin utilization genes by CcpA. J. Bacteriol. 176, 4527–4533 7913927 10.1128/jb.176.15.4527-4533.1994 PMC196271

[6] Castaño-Cerezo S., Bernal V., Blanco-Catalá J., Iborra J.L. and Cánovas M. (2011) cAMP-CRP co-ordinates the expression of the protein acetylation pathway with central metabolism in *Escherichia coli*. Mol. Microbiol. 82, 1110–1128 22059728 10.1111/j.1365-2958.2011.07873.x

[7] Castaño-Cerezo S., Bernal V., Post H., Fuhrer T., Cappadona S., Sánchez-Díaz N.C. et al. (2014) Protein acetylation affects acetate metabolism, motility and acid stress response in *Escherichia coli*. Mol. Syst. Biol. 10, 762 25518064 10.15252/msb.20145227 PMC4299603

[8] Liang W. and Deutscher M.P. (2012) Post-translational modification of RNase R is regulated by stress-dependent reduction in the acetylating enzyme Pka (YfiQ). RNA 18, 37–41 22124017 10.1261/rna.030213.111 PMC3261742

[9] Schilling B., Christensen D., Davis R., Sahu A.K., Hu L.I., Walker-Peddakotla A. et al. (2015) Protein acetylation dynamics in response to carbon overflow in *Escherichia coli*. Mol. Microbiol. 98, 847–863 26264774 10.1111/mmi.13161 PMC4715485

[10] Hentchel K.L., Thao S., Intile P.J. and Escalante-Semerena J.C. (2015) Deciphering the regulatory circuitry that controls reversible lysine acetylation in *Salmonella enterica*. mBio 6, e00891–e00915 26199328 10.1128/mBio.00891-15 PMC4513076

[11] Nambi S., Gupta K., Bhattacharyya M., Ramakrishnan P., Ravikumar V., Siddiqui N. et al. (2013) Cyclic AMP dependent protein lysine acylation in mycobacteria regulates fatty acid and propionate metabolism. J. Biol. Chem. 288, 14114–14124 23553634 10.1074/jbc.M113.463992 PMC3656268

[12] Xu J.-Y., You D., Leng P.-Q. and Ye B.-C. (2014) Allosteric regulation of a protein acetyltransferase in *Micromonospora aurantiaca* by the amino acids cysteine and arginine. J. Biol. Chem. 289, 27034–27045 25124041 10.1074/jbc.M114.579078 PMC4175341

[13] Berndsen C.E. and Denu J.M. (2005) Assays for mechanistic investigations of protein/histone acetyltransferases. Methods 36, 321–331 16085424 10.1016/j.ymeth.2005.03.002

[14] Kim Y, Tanner KG and Denu JM (2000) A continuous, nonradioactive assay for histone acetyltransferases. Anal. Biochem. 280, 308–314 10790315 10.1006/abio.2000.4546

[15] Wisniewski J.R., Zougman A., Naqaraj N. and Mann M. (2009) Universal sample preparation method for proteome analysis. Nat. Methods 6, 359–362 19377485 10.1038/nmeth.1322

[16] van den Berg M.A., de Jong-Gubbels P., Kortland C.J., van Kijken J.P., Pronk J.T. and Steensma H.Y. (1996) The two acetyl-coenzyme A synthetases of *Saccharomyces cerevisiae* differ with respect to kinetic properties and transcriptional regulation. J. Biol. Chem. 271, 28953–28959 8910545 10.1074/jbc.271.46.28953

[17] You D., Yao L.L., Huang D., Escalante-Semerena J.C. and Ye B.C. (2014) Acetyl-CoA synthetase is acetylated on multiple lysine residues by a protein acetyltransferase with single GNAT domain in *Saccharopolyspora erythraea*. J. Bacteriol. 196, 3169–3178 24957627 10.1128/JB.01961-14 PMC4135648

[18] Grant G.A. (2006) The ACT domain: a small molecule binding domain and its role as a common regulatory element. J. Biol. Chem. 281, 33825–33829 16987805 10.1074/jbc.R600024200

[19] Park S. and Imlay J.A. (2003) High levels of intracellular cysteine promote oxidative DNA damage by driving the fenton reaction. J. Bacteriol. 185, 1942–1950 12618458 10.1128/JB.185.6.1942-1950.2003 PMC150142

[20] Schuller D.J., Grant G.A. and Banaszak L.J. (1995) The allosteric ligand site in the V_max_-type cooperative enzyme phosphoglycerate dehydrogense. Nat. Struct. Biol. 2, 69–76 7719856 10.1038/nsb0195-69

[21] Siltberg-Liberles J. and Martinez A. (2009) Searching distant homologs of the regulatory ACT domain in phenylalanine hydroxylase. Amino Acids 36, 235–249 18368466 10.1007/s00726-008-0057-2

[22] Harman J.G. (2001) Allosteric regulation of the cAMP receptor protein. Biochim. Biophys. Acta 1547, 1–17 11343786 10.1016/s0167-4838(01)00187-x

[23] Zheng L., Xu T., Bai Z. and He B. (2014) Mn^2+^/Mg^2+^-dependent pyruvate kinase from a D-lactic acid-producing bacterium *Sporolactobacillus inulinus*: characterization of a novel Mn^2+^-mediated allosterically regulated enzyme. Appl. Microbiol. Biotechnol. 98, 1583–1593 23695776 10.1007/s00253-013-4907-9

[24] Bennett B.D., Kimball E.H., Gao M., Osterhout R., Van Dien S.J. and Rabinowitz J.D. (2009) Absolute metabolite concentrations and implied enzyme active site occupancy in *Escherichia coli*. Nat. Chem. Biol. 5, 593–599 19561621 10.1038/nchembio.186 PMC2754216

[25] Hentchel K.L. and Escalante-Semerena J.C. (2015) Acylation of biomolecules in prokaryotes: a widespread strategy for the control of biological function and metabolic stress. Microbiol. Mol. Biol. Rev. 79, 321–346 26179745 10.1128/MMBR.00020-15 PMC4503791

[26] Gardner J.G., Grundy F.J., Henkin T.M. and Escalante-Semerena J.C. (2006) Control of acetyl-coenzyme A synthetase (AcsA) activity by acetylation/deacetylation without NAD(+) involvement in *Bacillus subtilis*. J. Bacteriol. 188, 5460–5468 16855235 10.1128/JB.00215-06 PMC1540023

[27] Salinger A.J., Thoden J.B. and Holden H.M. (2016) Structural and functional investigation of FdhC from *acinetobacter nosocomialis*: a sugar N-acyltransferase belonging to the GNAT superfamily. Biochemistry 55, 4509–4518 27404806 10.1021/acs.biochem.6b00602 PMC5095703

[28] Favrot L., Blanchard J.S. and Vergnolle O. (2016) Bacterial GCN5-related N-acetyltransferases: from resistance to regulation. Biochemistry 55, 989–1002 26818562 10.1021/acs.biochem.5b01269 PMC4795176

[29] Crosby H.A., Pelletier D.A., Hurst G.B. and Escalante-Semerena J.C. (2012) System-wide studies of N-lysine acetylation in *Rhodopseudomonas palustris* reveal substrate specificity of protein acetyltransferases. J. Biol. Chem. 287, 15590–15601 22416131 10.1074/jbc.M112.352104 PMC3346091

[30] Tucker A.C. and Escalante-Semerena J.C. (2013) Acetoacetyl-CoA synthetase activity is controlled by a protein acetyltransferase with unique domain organization in *Streptomyces lividans*. Mol. Microbiol. 87, 152–167 23199287 10.1111/mmi.12088 PMC3535548

[31] Thao S. and Escalante-Semerena J.C. (2011) Biochemical and thermodynamic analyses of *Salmonella enterica* Pat, a multidomain, multimeric N(ε)-lysine acetyltransferase involved in carbon and energy metabolism. mBio 2, 1688–1698 10.1128/mBio.00216-11 PMC3195501 22010215

[32] de Diego Puente T., Gallego-Jara J., Castaño-Cerezo S., Bernal Sánchez V., Fernández Espín V., García de la Torre J. et al. (2015) The protein acetyltransferase PatZ from *Escherichia coli* is regulated by autoacetylation-induced oligomerization. J. Biol. Chem. 290, 23077–23093 26251518 10.1074/jbc.M115.649806 PMC4645606

[33] Han X., Shen L., Wang Q., Cen X., Wang J., Wu M. et al. (2017) Cyclic AMP inhibits the activity and promotes the acetylation of acetyl-CoA synthetase through competitive binding to the ATP/AMP pocket. J. Biol. Chem. 292, 1374–1384 27974467 10.1074/jbc.M116.753640 PMC5270480

[34] Nambi S., Basu N. and Visweswariah S.S. (2010) cAMP-regulated protein lysine acetylases in mycobacteria. J. Biol. Chem. 285, 24313–24323 20507997 10.1074/jbc.M110.118398 PMC2915667

[35] Xu H., Hegde S.S. and Blanchard J.S. (2011) Reversible acetylation and inactivation of *Mycobacterium tuberculosis* acetyl-CoA synthetase is dependent on cAMP. Biochemistry 50, 5883–5892 21627103 10.1021/bi200156t PMC3125470

[36] Liu X.X., Liu W.B. and Ye B.C. (2016) Regulation of a protein acetyltransferase in *Myxococcus xanthus* by the coenzyme NADP. J. Bacteriol. 198, 623–632 10.1128/JB.00661-15 PMC4751817 26598367

