# Supplementary material for: Identification and characterization of two types of amino acid-regulated acetyltransferases in actinobacteria
Source: Biosci Rep. 2017 Jul 4;37(4):BSR20170157. doi: 10.1042/BSR20170157 (PMC6434083; doi:10.1042/BSR20170157)
Supplement: Supplementary Figure S1-S3 and Table S1 [file BSR-2017-0157_supp.pdf]

Identification and characterization of two types of amino acid-regulated acetyltransferases in actinobacteria

Yu-Xing Lu[1], Xin-Xin Liu[1], Wei-Bin Liu[1], Bang-Ce Ye*[1,2]

[1]Lab of Biosystems and Microanalysis, Biomedical Nanotechnology Center, State Key Laboratory of Bioreactor Engineering, East China University of Science and Technology, Shanghai, 200237, China
[2]School of Chemistry and Chemical Engineering, Shihezi University, Xinjiang, 832000, China

Supporting Information

Table S1. Information of all PatA proteins in the InterPro database

NO.	UniProt Accession	Species	Length
1	A0A0J8A8R9	Streptomyces regensis	454
2	A0A0L8NL10	Streptomyces antibioticus	454
3	A0A0M8VZ48	Streptomyces sp. NRRL WC-3723	466
4	A0A0H4CE92	Streptomyces sp. PBH53	454
5	H2K515	Streptomyces hygroscopicus subsp. jinggangensis	454
6	A0A0N0TBJ4	Streptomyces sp. NRRL B-3648	470
7	A0A0N1GFH7	Actinobacteria bacterium OK074	486
8	L1KRU4	Streptomyces ipomoeae 91-03	445
9	A0A0J8BX62	Streptomyces viridochromogenes	473
10	A0A0M2MR32	Streptomyces sp. MUSC136T	452
11	A0A089X1X1	Streptomyces glaucescens	461
12	A0A0M8VRU3	Streptomyces sp. AS58	474
13	A0A0N0NC08	Actinobacteria bacterium OV320	458
14	S5VI80	Streptomyces collinus DSM 40733	479
15	A0A0G3AQ87	Streptomyces incarnatus	470
16	A0A0M8V759	Streptomyces sp. NRRL WC-3618	417
17	K4RDV7	Streptomyces davawensis JCM 4913	469
18	V6JMA3	Streptomyces roseochromogenus subsp. oscitans DS 12.976	457
19	A0A086GIX8	Streptomyces scabiei	437
20	C9ZB69	Streptomyces scabiei (strain 87.22)	356
21	A0A0L0KV36	Streptomyces stelliscabiei	434
22	M3G1M0	Streptomyces bottropensis ATCC 25435	434
23	A0A066Y5P7	Streptomyces olindensis	411
24	A0A0M8X1H4	Streptomyces sp. NRRL B-1140	418
25	A0A0K8PK97	Streptomyces azureus	422

26	D9XGL8	Streptomyces viridochromogenes DSM 40736	417
27	S4MKI0	Streptomyces afghaniensis 772	359
28	A0A0L8LKK4	Streptomyces resistomycificus	428
29	A0A0M2Z3S5	Streptomyces sp. MUSC119T	463
30	A0A0F7W7I5	Streptomyces leeuwenhoekii	473
31	F3NTL4	Streptomyces griseoaurantiacus M045	478
32	A0A0M2JHK1	Streptomyces sp. MUSC149T	456
33	D9UU68	Streptomyces sp. (strain SPB78)	469
34	F3ZB44	Streptomyces sp. Tu6071	457
35	D6M4N3	Streptomyces sp. (strain SPB074)	475
36	A0A0H1AX99	Streptomyces sp. KE1	447
37	D6B750	Streptomyces albus J1074	447
38	M9T228	Streptomyces albus J1074	500
39	V4IRJ6	Streptomyces sp. PVA 94-07	444
40	A0A022M9D8	Streptomyces sp. Tu 6176	471
41	A3KKM3	Streptomyces ambofaciens ATCC 23877	451
42	S2YE05	Streptomyces sp. HGB0020	450
43	A0A086N6F4	Streptomyces mutabilis	450
44	A0A076MEA2	Streptomyces lividans TK24	452
45	Q93IX7	Streptomyces coelicolor ATCC BAA-471	452
46	A0A0M9X8D4	Streptomyces caelestis	419
47	A0A072SLP1	Streptomyces griseorubens	419
48	M3DX50	Streptomyces gancidicus BKS 13-15	419
49	A0A081XQT0	Streptomyces toyocaensis	419
50	D5ZV95	Streptomyces ghanaensis ATCC 14672	431
51	A0A059W850	Streptomyces albulus	467
52	A0A0A8ECX2	Streptomyces sp. 769	470
53	A0A0L8M3H2	Streptomyces decoyicus	465
54	J1RX27	Streptomyces auratus AGR0001	441
55	A0A0D4DF88	Streptomyces lydicus A02	463
56	A0A0N1JW68	Streptomyces chattanoogensis	463
57	A0A0D7CN04	Streptomyces natalensis ATCC 27448	468
58	A0A0L8N0W4	Streptomyces griseoflavus	463
59	A0A0M8RKB1	Streptomyces sp. NRRL F-5755	463
60	A0A0L8PYV2	Streptomyces aureofaciens	463
61	A0A0M9XDE9	Streptomyces rimosus subsp. rimosus	463
62	A0A0M9XT07	Streptomyces rimosus subsp. pseudoverticillatus	463
63	A0A0F4IC38	Streptomyces sp. NRRL S-104	485
64	A0A0M8U8A4	Streptomyces sp. H021	485
65	A0A024YIA4	Streptomyces sp. PCS3-D2	484
66	A0A0N0ST97	Streptomyces sp. H036	484
67	A0A0F0HGM5	Streptomyces sp. NRRL F-4428	478
68	A0A0L8MX44	Streptomyces virginiae	486
69	A0A0M8RV45	Streptomyces sp. WM6368	483
70	A0A0J6XSP8	Streptomyces roseus	477
71	A0A0G3UQN3	Streptomyces sp. Mg1	490
72	B4V827	Streptomyces sp. Mg1	458
73	A0A0M8SQD7	Streptomyces sp. WM4235	486
74	A0A0M9CL64	Streptomyces sp. XY332	489

75	A0A0M9Y5R8	Streptomyces sp. WM6372	475
76	A0A0N0MU20	Actinobacteria bacterium OV450	497
77	A0A0C2AEL5	Streptomyces sp. AcH 505	451
78	A0A0M8T7Q6	Streptomyces sp. WM6378	465
79	A0A0C2B5K8	Streptomyces sp. 150FB	451
80	E2Q9K6	Streptomyces clavuligerus DSM 738	488
81	A0A0B5HPE5	Streptomyces vietnamensis	481
82	F2RA54	Streptomyces venezuelae DSM 40230	488
83	A0A0L8KAD7	Streptomyces viridochromogenes	490
84	A0A0M8YH92	Streptomyces sp. NRRL F-6491	477
85	A0A0M7R2A2	Streptomyces venezuelae	490
86	B5H7N9	Streptomyces pristinaespiralis ATCC 25486	467
87	A0A087K1P7	Streptomyces sp. JS01	475
88	A0A0D6VIE0	Streptomyces griseus	473
89	D6AIK2	Streptomyces roseosporus NRRL 15998	450
90	A0A0M5ISK3	Streptomyces sp. CFMR 7	450
91	N0CLA4	Streptomyces fulvissimus DSM 40593	471
92	A0A069JPG3	Streptomyces sp. NTK 937	535
93	G2NNI8	Streptomyces sp. (strain SirexAA-E / ActE)	460
94	A0A0N1K5W2	Streptomyces sp. NRRL S-4	482
95	E8WCA9	Streptomyces pratensis ATCC 33331	506
96	M9TRH0	Streptomyces sp. PAMC26508	503
97	I2MUR9	Streptomyces tsukubaensis NRRL18488	493
98	D9WFP9	Streptomyces himastatinicus ATCC 53653	405
99	D7BY67	Streptomyces bingchenggensis (strain BCW-1)	418
100	G2P3F2	Streptomyces violaceusniger Tu 4113	420
101	A0A014L396	Streptomyces sp. PRh5	422
102	A0A0A0N8V9	Streptomyces rapamycinicus NRRL 5491	432
103	A0A060ZFA1	Streptomyces iranensis	427
104	A0A0F5AAC7	Streptomyces sp. MUSC164	429
105	M3CC83	Streptomyces mobaraensis DSM 40847	464
106	G8WNJ2	Streptomyces cattleya DSM 46488	496
107	A0A0N0H377	Streptomyces sp. NRRL F-6602	409
108	A0A081EMD0	Streptomyces fradiae	484
109	A0A0F4J786	Streptomyces sp. NRRL S-495	371
110	A0A0M8TZX7	Streptomyces sp. XY431	470
111	E4N654	Kitasatospora setae DSM 43861	456
112	A0A066Z0R8	Kitasatospora cheerisanensis KCTC 2395	446
113	A0A0D0PVV0	Kitasatospora griseola	444
114	A4FK80	Saccharopolyspora erythraea DSM 40517	444
115	G8SFD4	Actinoplanes sp. ATCC 31044	347
116	I0HG80	Actinoplanes missouriensis DSM 43046	356
117	A0A0A6UKE2	Actinoplanes utahensis	340
118	R4LY11	Actinoplanes sp. N902-109	355
119	U5WB92	Actinoplanes friuliensis DSM 7358	356
120	D9SZU3	Micromonospora aurantiaca DSM 43813	362
121	A0A0N0AJ72	Micromonospora sp. NRRL B-16802	354
122	I0KZ58	Micromonospora lupini str. Lupac 08	351
123	F4FEZ5	Verrucosispora maris AB-18-032	351

124	A0A0D0X4H6	Micromonospora carbonacea	354
125	C4REH0	Micromonospora sp. ATCC 39149	379
126	A0A0C2JDG3	Streptomonospora alba	364
127	W2F2X9	Microbispora sp. ATCC PTA-5024	387
128	D2AVA5	Streptosporangium roseum DSM 43021	344
129	D6Y1U2	Thermobispora bispora DSM 43833	345
130	A0A0A1DIU0	Nocardioides simplex	289
131	F4CS81	Pseudonocardia dioxanivorans DSM 44775	356
132	A0A0F2GAU6	Nocardioides luteus	303
133	E9V0H2	Nocardioidaceae bacterium Broad-1	303
134	A0A0M8YNU3	Saccharothrix sp. NRRL B-16348	360
135	A0A0N0T6Y4	Nocardia sp. NRRL S-836	340
136	C6WC99	Actinosynnema mirum DSM 43827	372
137	A0A0K3B974	Kibdelosporangium sp. MJ126-NF4	350
138	A0A0H5CLW2	Alloactinosynnema sp. L-07	357
139	W7J5M6	Actinokineospora spheciospongiae	361
140	A0A0C1NNK1	Prauserella sp. Am3	392
141	G0G662	Amycolatopsis mediterranei strain S699	353
142	R1HIU7	Amycolatopsis vancoresmycina DSM 44592	354
143	A0A066TXE0	Amycolatopsis rifamycinica	353
144	A0A076N2W9	Amycolatopsis methanolica 239	358
145	A0A093BA80	Amycolatopsis lurida NRRL 2430	361
146	M2YM44	Amycolatopsis decaplanina DSM 44594	372
147	M2QMD0	Amycolatopsis azurea DSM 43854	373
148	A0A075V0T2	Amycolatopsis japonica	361
149	A0A094MMF1	Amycolatopsis sp. MJM2582	361
150	R4T1Q3	Amycolatopsis orientalis HCCB10007	361

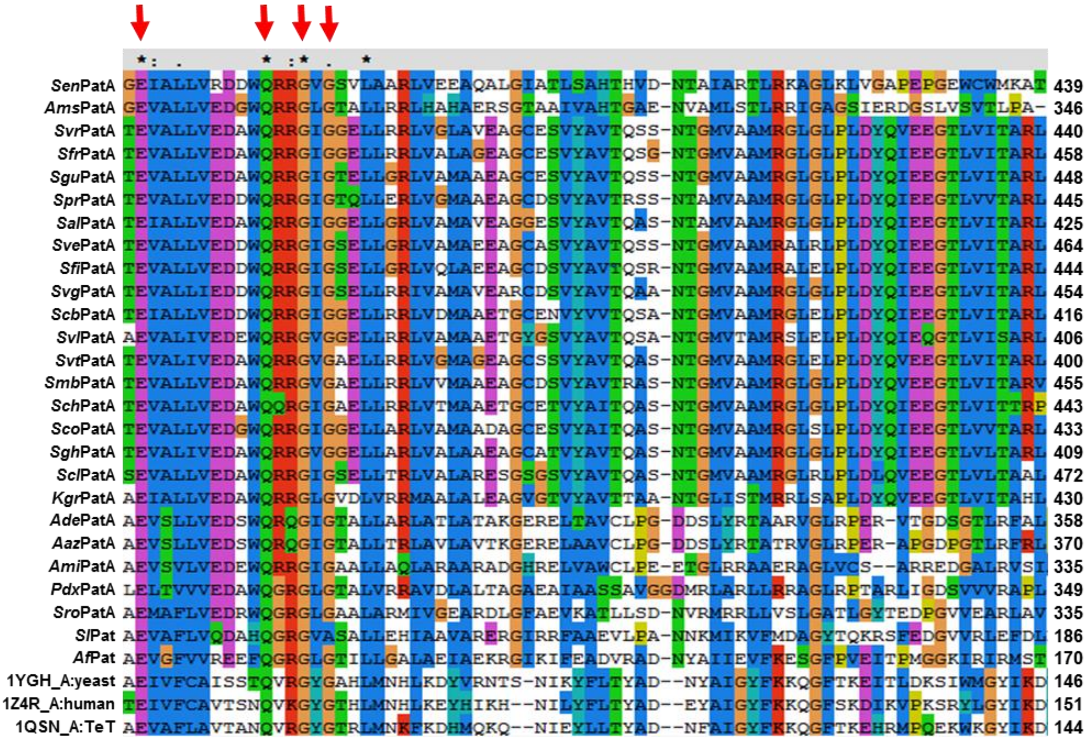

Figure S1. The multiple sequence alignment of GNAT domains of 24 AAPatA with other other protein acetyltransferases in prokaryotes and eukaryotes, including 1Z4RA in human beings, 1QSNA in *tetrahymena*, 1YGH_A in yeast, *Sl*Pat in *S. lividans*, and *Af*Pat in *Acidimicrobium ferrooxidans.* The red arrows indicated a conserved glutamate as a catalytic base, and a conserved motif sequence QXXGX(G/A) for acetyl-CoA recognition and binding.

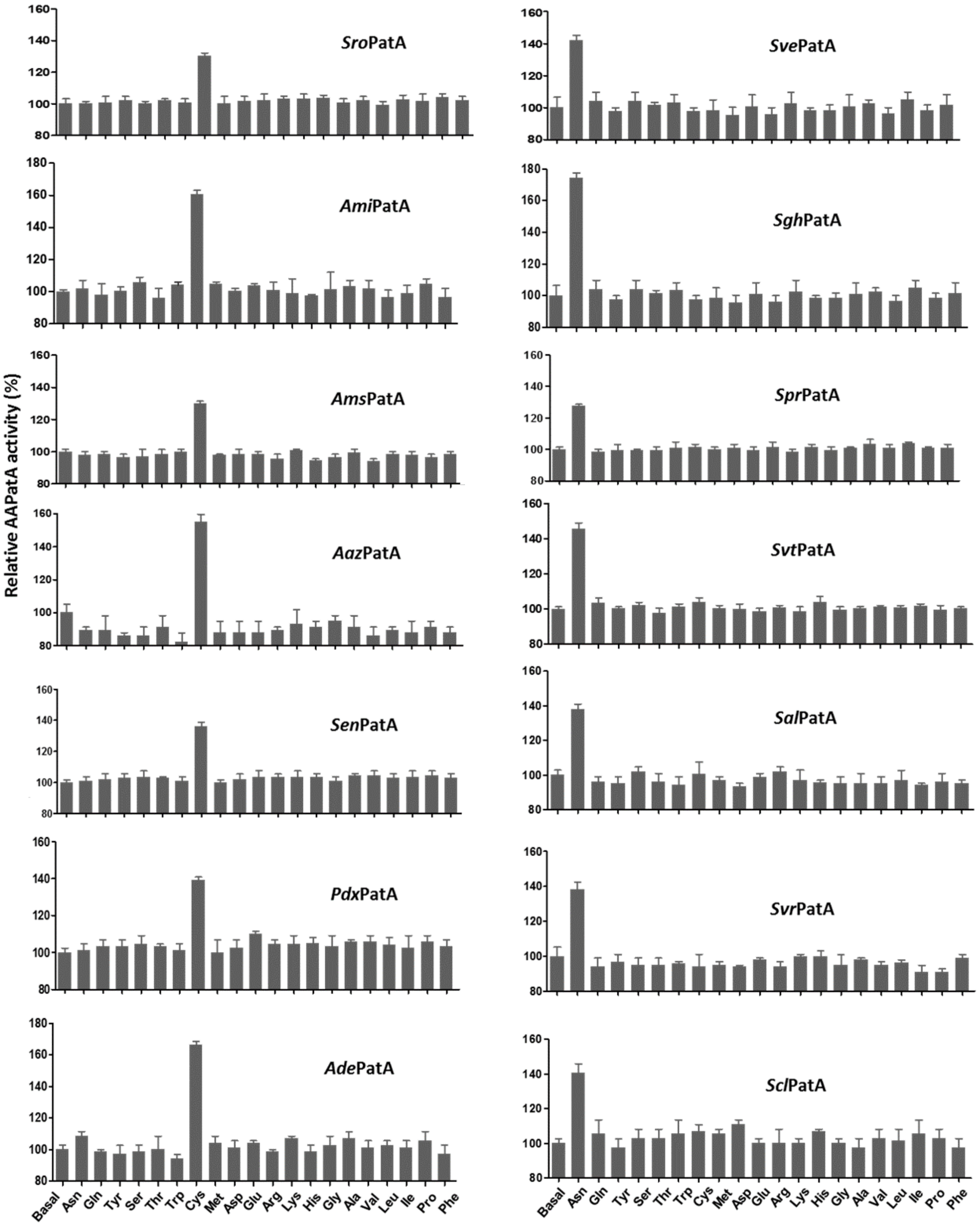

Figure S2. The allosteric effects of the ACT domain on acetylation activity in response to all amino acids. Acetylation activity of acetyltransferases was measured continuously by a coupled enzymatic assay using a fluorescence plate reader (Bio-Tek Instrument, Winooski, USA). The production of CoA is measured using pyruvate dehydrogenase-mediated reduction of NAD^+ to NADH, resulting in an increase of absorbance at 340 nm (ΔA_{340}). The putative AMP-forming acetyl-CoA synthetase *Ami*Acs from *Actinosynnema mirum* strain DSM 43827 was used as the substrate. *Svr*PatA from *Streptomyces viridochromogenes*, *Sgu*PatA from *Streptomyces glaucescens*, *Scb*PatA from *Streptomyces scabiei, Svt*PatA from *Streptomyces viridochromogenes*, *Sal*PatA from *Streptomyces albus* J1074, *Sco*PatA from *Streptomyces coelicolor*, *Sgh*PatA from *Streptomyces ghanaensis*, *Sch*PatA from *Streptomyces chattanoogensis*, *Svg*PatA from *Streptomyces virginiae*, *Scl*PatA from *Streptomyces clavuligerus*, *Sve*PatA from *Streptomyces venezuelae*, *Sfi*PatA from *Streptomyces fulvissimus*, *Svl*PatA from *Streptomyces violaceusniger*, *Spr*PatA from *Streptomyces pristinaespiralis*, *Smb*PatA from *Streptomyces mobaraensis*, *Sfr*PatA

from *Streptomyces fradiae*, *Kgr*PatA from *Kitasatospora griseola*, *Sen*PatA from *Saccharopolyspora erythraea*, *Ams*PatA from *Actinoplanes missouriensis*, *Sro*PatA from *Streptosporangium roseum*, *Pdx*PatA from *Pseudonocardia dioxanivorans*, *Ami*PatA from *Actinosynnema mirum*, *Ade*PatA from A*mycolatopsis decaplanina*, *Aaz*PatA from *Amycolatopsis azurea*.

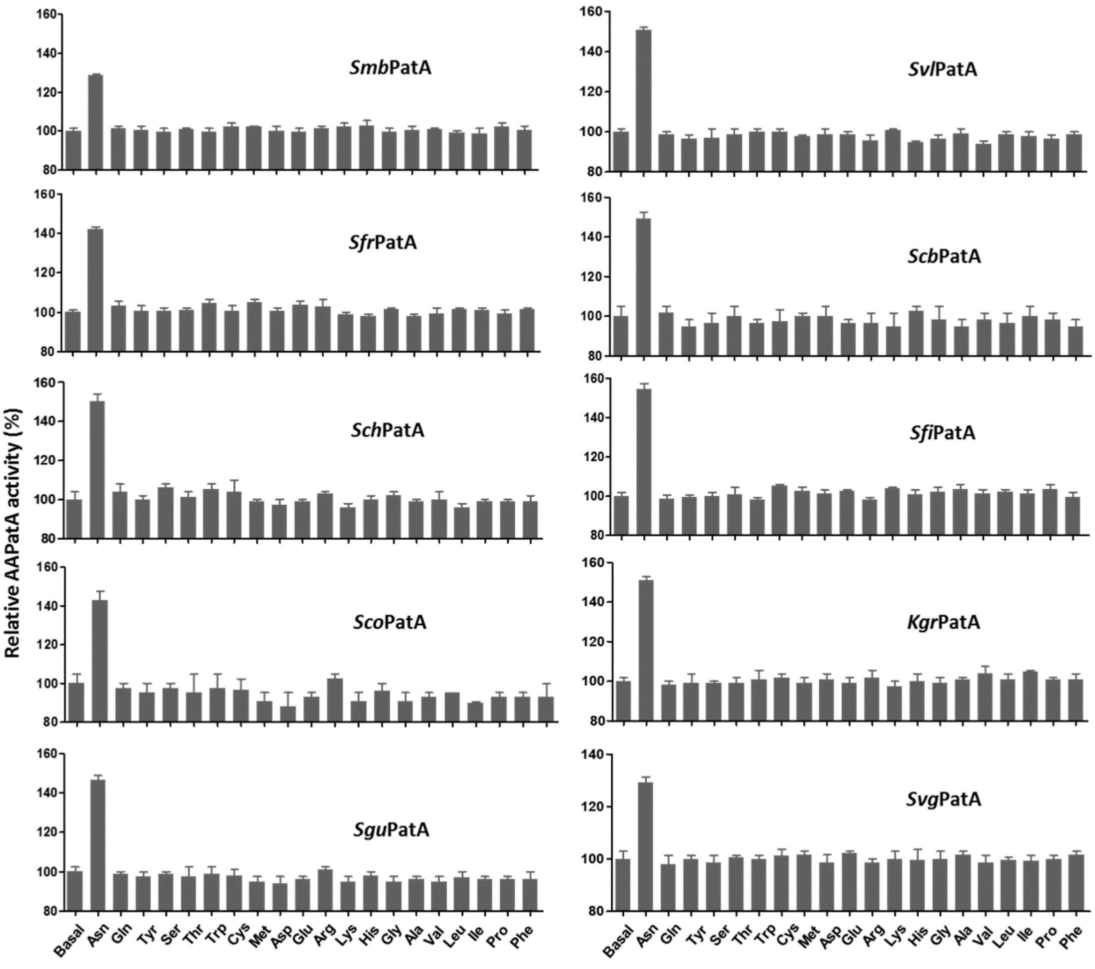

Figure S2 (continuing). The allosteric effects of the ACT domain on acetylation activity in response to all amino acids.

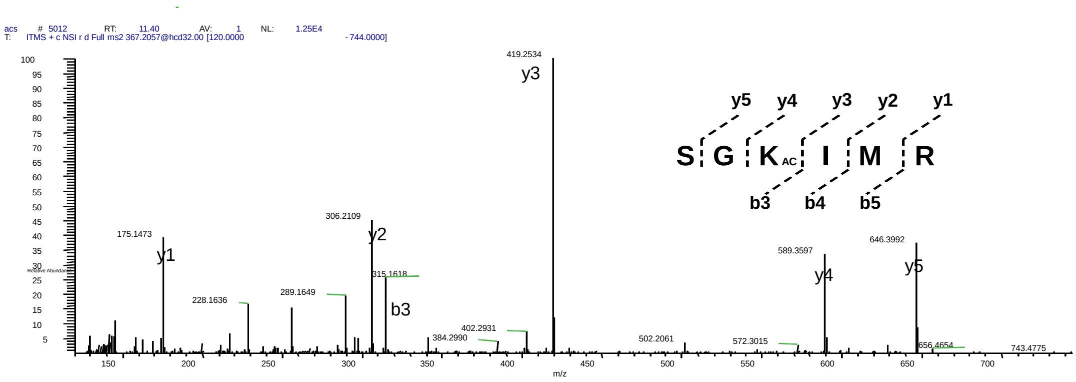

Figure S3. The MS spectra of the acetylpeptide from in vitro acetylated *Ami*Acs protein by *Ami*PatA acetyltransferase. SGKIMR is located from 617th amino acid residue to the 623rd amino acid residue in *Ami*Acs
